# Genomic Surveillance for SARS-CoV-2 Variants: Predominance of the Delta (B.1.617.2) and Omicron (B.1.1.529) Variants — United States, June 2021–January 2022

**DOI:** 10.15585/mmwr.mm7106a4

**Published:** 2022-02-11

**Authors:** Anastasia S. Lambrou, Philip Shirk, Molly K. Steele, Prabasaj Paul, Clinton R. Paden, Betsy Cadwell, Heather E. Reese, Yutaka Aoki, Norman Hassell, Xiao-yu Zheng, Sarah Talarico, Jessica C. Chen, M. Steven Oberste, Dhwani Batra, Laura K. McMullan, Alison Laufer Halpin, Summer E. Galloway, Duncan R. MacCannell, Rebecca Kondor, John Barnes, Adam MacNeil, Benjamin J. Silk, Vivien G. Dugan, Heather M. Scobie, David E. Wentworth, Strain Surveillance and Emerging Variants Bioinformatic Working Group, Strain Surveillance and Emerging Variants NS3 Working Group

**Affiliations:** ^1^CDC COVID-19 Emergency Response Team; ^2^Epidemic Intelligence Service, CDC; ^3^General Dynamics Information Technology, Falls Church, Virginia; ^4^Office of Advanced Molecular Detection, National Center for Emerging and Zoonotic Infectious Diseases, CDC.

Genomic surveillance is a critical tool for tracking emerging variants of SARS-CoV-2 (the virus that causes COVID-19), which can exhibit characteristics that potentially affect public health and clinical interventions, including increased transmissibility, illness severity, and capacity for immune escape. During June 2021–January 2022, CDC expanded genomic surveillance data sources to incorporate sequence data from public repositories to produce weighted estimates of variant proportions at the jurisdiction level and refined analytic methods to enhance the timeliness and accuracy of national and regional variant proportion estimates. These changes also allowed for more comprehensive variant proportion estimation at the jurisdictional level (i.e., U.S. state, district, territory, and freely associated state). The data in this report are a summary of findings of recent proportions of circulating variants that are updated weekly on CDC’s COVID Data Tracker website to enable timely public health action.^†^ The SARS-CoV-2 Delta (B.1.617.2 and AY sublineages) variant rose from 1% to >50% of viral lineages circulating nationally during 8 weeks, from May 1–June 26, 2021. Delta-associated infections remained predominant until being rapidly overtaken by infections associated with the Omicron (B.1.1.529 and BA sublineages) variant in December 2021, when Omicron increased from 1% to >50% of circulating viral lineages during a 2-week period. As of the week ending January 22, 2022, Omicron was estimated to account for 99.2% (95% CI = 99.0%–99.5%) of SARS-CoV-2 infections nationwide, and Delta for 0.7% (95% CI = 0.5%–1.0%). The dynamic landscape of SARS-CoV-2 variants in 2021, including Delta- and Omicron-driven resurgences of SARS-CoV-2 transmission across the United States, underscores the importance of robust genomic surveillance efforts to inform public health planning and practice. 

In November 2020, CDC expanded its genomic surveillance program to track SARS-CoV-2 lineages at the national and U.S. Department of Health and Human Services (HHS) regional levels (*1*,*2*). CDC also initiated SARS-CoV-2 Sequencing for Public Health Emergency Response, Epidemiology, and Surveillance^§^ (SPHERES), a national SARS-CoV-2 genomic surveillance consortium. Currently, the national genomic surveillance program integrates three principal sources of SARS-CoV-2 sequence data: 1) the National SARS-CoV-2 Strain Surveillance (NS3) program^¶^; 2) CDC-contracted commercial sequencing data; and 3) sequences from public health, academic, and clinical laboratories that are tagged** as baseline surveillance in public genomic data repositories, such as Global Initiative on Sharing All Influenza Data (GISAID) and National Center for Biotechnology Information (NCBI) GenBank. Inclusion of tagged SARS-CoV-2 sequence data was instituted in October 2021 to enhance the geographic representativeness and precision of variant proportion estimates and to enhance the surveillance program’s sustainability.

SARS-CoV-2 consensus sequences^††^ submitted or tagged for national genomic surveillance were combined, assessed for quality, deduplicated, and analyzed for weekly estimation of variant proportions at the national, HHS regional, and jurisdictional levels. SARS-CoV-2 variant proportions (with 95% CIs) were estimated weekly for variants of concern, variants of interest, variants being monitored,^§§^ and any other lineages accounting for >1% of sequences nationwide during the preceding 12 weeks. Proportion estimation methods used a complex survey design with statistical weights to correct potential biases because samples selected for sequencing might not be representative of all SARS-CoV-2 infections (Box).^¶¶^ Each submitting laboratory source was considered a primary sampling unit, and the geographic level (i.e., jurisdictional, HHS regional, or national) and week of sample collection for each sequence, a stratum. Weights account for the probability that a sample from an infection is sequenced and are trimmed to the 99th percentile. Variant proportion estimates that did not meet the National Center for Health Statistics’ data presentation standards for proportions were flagged.*** During June 2021–January 2022, the median interval from SARS-CoV-2 sample collection to availability of consensus sequences was 15 days. Therefore, to estimate variant proportions during the most recent 2 weeks, multinomial regression models were fit for national and regional estimates to nowcast (*2*) variant proportions with corresponding 95% projection intervals^†††^ using the most recent 21 weeks of data for prediction. To compare the speeds of initial variant transmission, the doubling time of each variant was calculated using the “time” covariate in nowcast models. All analyses used PANGO SARS-CoV-2 lineage nomenclature and sublineages were aggregated under the parent lineage (*3*). This activity was reviewed by CDC and conducted consistent with applicable federal law and CDC policy.^§§§^

BOX SARS-CoV-2 variant* proportion estimation methods,^†^ — United States, June 2021–January 2022

**Estimated weighted proportions: weighted analysis using complex survey design methods to produce weekly estimates**

**Survey design**

- Primary sampling unit
  - Laboratory source of the sequence
- Strata
  - Geography (region/jurisdiction) and week
- Analysis weights
  - Number of infections represented by each sequence
  - Adjusted for known oversampling of S-gene target failure (SGTF) specimens
  - Weights greater than 99th percentile are trimmed and redistributed

**Variants included**

- Variant of concern
- Variant of interest
- Variant being monitored
- >1% of unweighted sequences in the 12 weeks before the most recent 2 weeks

**Geographic level of analysis**

- Jurisdiction
- U.S. Department of Health and Human Services (HHS) region
- National

**Period**

- Jurisdictions: variant proportions for the combined 4 weeks preceding the most recent 2 weeks
- HHS Region and national: weekly variant proportions for the past 3–12 weeks

**Nowcast model: multinomial regression analysis of complex survey data**

**Survey design**

- Primary sampling unit
  - Laboratory source of the sequence
- Strata
  - Geography (region/jurisdiction) and week
- Analysis weights
  - Number of infections represented by each sequence
  - Adjusted for known oversampling of SGTF specimens
  - Weights greater than 99th percentile are trimmed and redistributed

**Variants included**

- Variant of concern
- Variant of interest
- Variant being monitored
- >1% of unweighted sequences in the 12 weeks before the most recent 2 weeks

**Geographic level of analysis**

- HHS region
- National

**Period**

- Weekly variant proportions for the most recent 2 weeks

* https://www.cdc.gov/coronavirus/2019-ncov/cases-updates/variant-surveillance/variant-info.html

^†^ https://github.com/CDCgov/SARS-CoV-2_Genomic_Surveillance

Genomic sequencing capacity in the United States has increased in both throughput and participating laboratories during the COVID-19 pandemic, with 1,189,459 sequences submitted during June 2021–January 2022. The corresponding average of 35,431 sequences per week is approximately three times higher than the 10,643 sequences per week during the surveillance period covered by the previous report (December 2020–May 2021) (*2*). As of the week ending January 22, 2022, a total of 1,469,400 SARS-CoV-2 sequences met the criteria^¶¶¶^ for being included in national genomic surveillance estimates; 88% of sequences were from CDC-contracted commercial diagnostic laboratories, 2% from NS3, and 10% were baseline-tagged sequences. Sequences originated from 56 jurisdictions: 50 U.S. states, District of Columbia, American Samoa, Guam, Northern Mariana Islands, Puerto Rico, and U.S. Virgin Islands.

During June 2021, the proportion of several variants changed markedly (Figure 1). Alpha (B.1.1.7 and Q sublineages) continued to decline nationally. Gamma (P.1 and descendent lineages) peaked at 12.1% (95% CI = 9.8%–14.7%) during the week ending June 5, 2021, before declining; Mu (B.1.621) and Lambda (C.37) increased to their peaks of 4.5% (95% CI = 3.5%–5.6%) and 0.6% (95% CI = 0.3%–0.9%), respectively, for the week ending June 19, before declining as Delta (B.1.617.2 and AY sublineages) reached predominance (>50%).**** The overall effect was a reduction in SARS-CoV-2 variant diversity because of Delta’s growth in proportion, with five variants being monitored circulating at >1% in June and only one variant circulating above this threshold in September. The Delta variant rose from 1% of circulating SARS-CoV-2 viruses nationally during the week ending May 1, to >50% by the week ending June 26, and to >95% by the week ending July 31. Delta prevalence was >95% in all 10 HHS regions^††††^ by the week ending July 31 and remained >50% in each region for ≥24 weeks.

**FIGURE 1:**
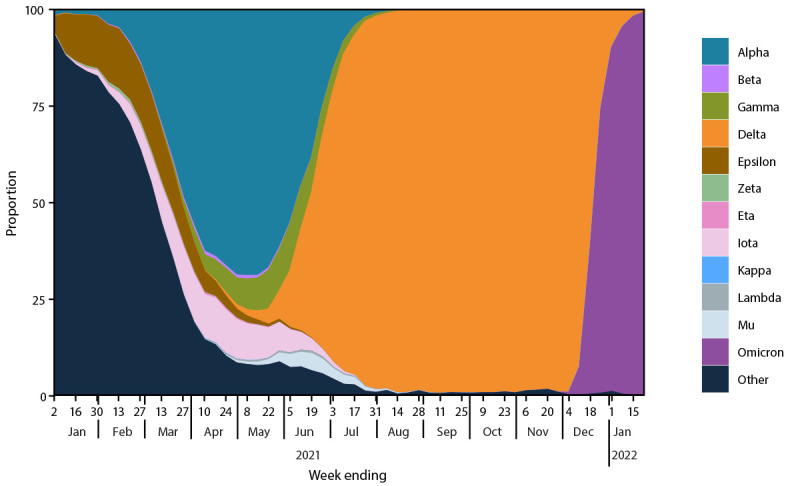
National weekly proportion estimates* of SARS-CoV-2 variants^†^ — United States, January 2, 2021–January 22, 2022 **Abbreviations**: NS3 = National SARS-CoV-2 Strain Surveillance program; PANGO = Phylogenetic Assignment of Named Global Outbreak; WHO = World Health Organization. * Sequences are reported to CDC through NS3, contract laboratories, public health laboratories, and other U.S. institutions. Variant proportion estimation methods use a complex survey design and statistical weights to account for the probability that a specimen is sequenced. ^†^ SARS-CoV-2 WHO variant label and PANGO lineage: Alpha (B.1.1.7); Beta (B.1.351); Gamma (P.1); Delta (B.1.617.2), Epsilon (B.1.427/B.1.429); Zeta (P.2); Eta (B.1.525); Iota (B.1.526); Kappa (B.1.617.1); Lambda (C.37); Mu (B.1.621); and Omicron (B.1.1.529). https://www.cdc.gov/coronavirus/2019-ncov/variants/variant-classifications.html

The Omicron variant proportion rapidly increased after the first U.S. case was reported on December 1 (*4*). Omicron first accounted for >1% of circulating lineages nationally during the week ending December 11, 2021, >50% of viruses for the week ending December 25, and >95% by the week ending January 8, 2021. As of the week ending January 22, 2022, national genomic surveillance estimates were 99.2% (95% CI = 99.0%–99.5%) for Omicron and 0.7% (95% CI = 0.5%–1.0%) for Delta. Region 7 had the highest proportion of Delta (3.0%; 95% CI = 1.9%–4.4%) and the lowest proportion of Omicron (97.0%; 95% CI =95.6%–98.1%). Region 9 had the highest proportion of Omicron (99.8%; 95% CI = 99.6%–99.9%) and the lowest proportion of Delta (0.2%; 95% CI = 0.1%–0.4%). Omicron’s variant proportion had an estimated initial doubling time of 3.2 days (95% CI = 3.1–3.4 days), which was faster than those of Delta (7.2 days; 95% CI = 7.0–7.4 days), Alpha (11.0 days; 95% CI = 8.3–16.1 days), Gamma (13.1 days; 95% CI = 12.0–14.3 days), and Mu (14.7 days; 95% CI = 13.8–15.7 days). Omicron rose from 1% to 99% of infections nationally in 6 weeks, compared with 18 weeks for Delta (Figure 2).

**FIGURE 2:**
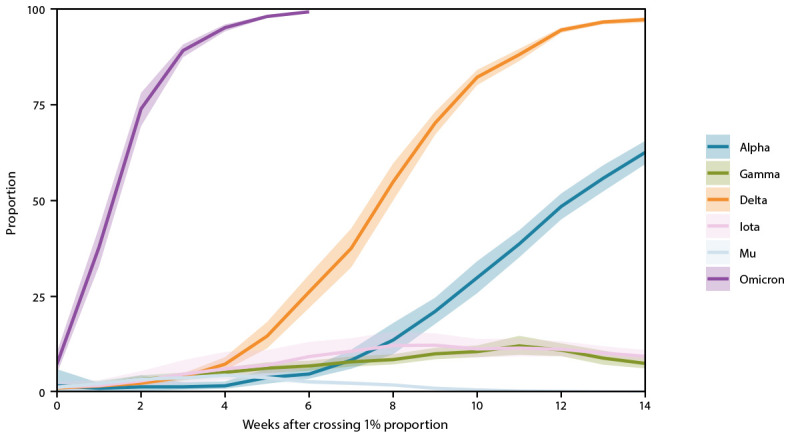
Estimated variant proportions with 95% confidence intervals* during the first 14 weeks of each variant’s emergence (from the time of exceeding 1% of national circulating viruses) for six SARS-CoV-2 variants^†^ — United States, November 2020–January 2022 **Abbreviations**: NS3 = National SARS-CoV-2 Strain Surveillance program; PANGO = Phylogenetic Assignment of Named Global Outbreak; WHO = World Health Organization. * 95% CIs for estimates are shown by shaded areas. Sequences are reported to CDC through NS3, contract laboratories, public health laboratories, and other U.S. institutions. The methods for estimating variant proportions and 95% CIs use a complex survey design and statistical weights to account for the probability that a specimen is sequenced. ^†^ SARS-CoV-2 WHO variant label and PANGO lineage: Alpha (B.1.1.7), Beta (B.1.351), Gamma (P.1), Delta (B.1.617.2), Mu (B.1.621), and Omicron (B.1.1.529). https://www.cdc.gov/coronavirus/2019-ncov/variants/variant-classifications.html

## Discussion

This report summarizes CDC’s weekly surveillance of variant proportions, which are used to drive public health action. The proportional distribution of SARS-CoV-2 variants circulating in the United States changed considerably during 2021. In spring 2021, Alpha co-circulated nationally with several other variants (e.g., Gamma, Delta, Eta, and Iota), but Delta became the predominant variant nationally in late June. Delta remained the only SARS-CoV-2 variant circulating at a high proportion from August–November but was rapidly overtaken by Omicron in late December. The rises of the Delta and Omicron variants were associated with major surges in COVID-19 cases during July–September 2021 and December 2021–January 2022, respectively.^§§§§^ The Omicron-driven wave that started in December 2021 is declining. These variant dynamics illustrate how SARS-CoV-2 has continued to evolve, with different variants defining different phases of the COVID-19 pandemic.

Variant emergence and growth are likely influenced by a combination of viral and host population factors. Factors contributing to Delta’s rise in prevalence include increased transmissibility and a subtle increase in immune escape relative to previous variants (*5*,*6*). Omicron’s rise in prevalence was likely driven by increased transmissibility (*7*) that might be due primarily to immune escape^¶¶¶¶^ (*8*), which also decreases the effectiveness of vaccines and monoclonal antibodies (*9*). However, early studies suggest that the relative severity of disease attributed to Omicron infections is lower than that resulting from infections with other SARS-CoV-2 variants.*****^,^^†††††^ A variant’s ability to spread and cause disease is affected by population susceptibility (duration of variant-specific immunity, cross-protection from previous infections, and vaccine-induced immunity). Transmission is also influenced by human behavior, particularly through prevention strategies.

The findings of this report are subject to at least four limitations. First, estimates might be biased by nonrandom sampling of specimens or differential timing of reporting (e.g., prioritizing sequences with S-gene target failure^§§§§§^ or sequences from international travelers). Second, the precision of estimates of newly emerging variants is initially affected by relatively small numbers of available sequences, especially at the jurisdictional and regional levels. Third, current variant estimation analyses might differ from past analyses because of changes in PANGO lineage definitions over time. Finally, the presented dates correspond to clinical testing, and noted variants were likely present before these periods; for example, wastewater surveillance indicates that Omicron was circulating in the United States >1 week before the first reported case (*10*).

CDC’s national SARS-CoV-2 genomic surveillance program has expanded data sources and refined analytic methods to enhance the timeliness and accuracy of national and regional variant proportion estimates. These changes have enhanced the robustness and representativeness of variant proportion estimates. Nowcast modeling at multiple geographic levels has enabled more timely estimation and demonstrated an ability to monitor emerging variants, even those circulating at low levels. SARS-CoV-2 variants are expected to continue emerging; a future variant might challenge the predominance of Omicron and exhibit different characteristics that affect public health and clinical interventions. Consequently, it is important to maintain SARS-CoV-2 genomic surveillance to ensure emerging variants are monitored and to promptly inform public health planning and practice.

**Summary**

**What is already known about this topic?**

CDC conducts genomic surveillance to track SARS-CoV-2 variants in the United States.

**What is added by this report?**

CDC’s SARS-CoV-2 genomic surveillance has been expanded to incorporate sequence data from public repositories and to produce weighted estimates of variant proportions at the jurisdiction level. The Delta (B.1.617.2 and AY sublineages) variant rose to predominance in late June 2021, followed by the rapid rise of Omicron (B.1.1.529 and BA sublineages) in December 2021.

**What are the implications for public health practice?**

The dynamic landscape of SARS-CoV-2 variants in 2021, including Delta- and Omicron-driven resurgences of SARS-CoV-2 transmission across the United States, underscores the importance of robust genomic surveillance efforts to inform public health planning and practice.

## Footnotes

* These authors contributed equally to this report.

† Estimates on CDC COVID Data Tracker may vary slightly from those in this report because the estimates were calculated on different days. https://covid.cdc.gov/covid-data-tracker/#variant-proportions

§ https://www.cdc.gov/coronavirus/2019-ncov/variants/spheres.html

¶
https://www.aphl.org/programs/preparedness/Crisis-Management/COVID-19-Response/Pages/Sequence-Based-Surveillance-Submission.aspx

** Sequence tagging allows for sequencing partners to tag or label randomly sampled SARS-CoV-2 sequences submitted via GISAID EpiCov and NCBI GenBank to be used in CDC genomic surveillance estimates. https://www.aphl.org/programs/preparedness/Crisis-Management/Documents/Technical-Assistance-for-Categorizing-Baseline-Surveillance-Update-Oct2021.pdf

†† A consensus sequence is produced by aligning SARS-CoV-2 nucleotide sequences produced through sequencing a sample and then determining the most common nucleotide at each position. It is an interoperable genomic surveillance unit that can be combined from laboratory sources.

§§
https://www.cdc.gov/coronavirus/2019-ncov/cases-updates/variant-surveillance/variant-info.html

¶¶
https://github.com/CDCgov/SARS-CoV-2_Genomic_Surveillance

*** Flagged estimates are presented with a note indicating they might be less reliable. https://www.cdc.gov/nchs/data/series/sr_02/sr02_175.pdf

††† CIs show uncertainty around an estimate describing observed data; prediction intervals show uncertainty around predictions of unobserved data, such as the nowcast variant proportions.

§§§ 45 C.F.R. part 46.102(l)(2), 21 C.F.R. part 56; 42 U.S.C. Sect.241(d); 5 U.S.C.0 Sect.552a; 44 U.S.C. Sect. 3501 et seq.

¶¶¶ Sequences are first excluded if they are not assigned a PANGO lineage, and then are filtered to include only human hosts and U.S.-specific sequences. This pool of sequences is then deduplicated, and finally, sequences with invalid state names, laboratory sources, and weights are dropped.

**** Predominance refers to a variant accounting for >50% of national circulating SARS-CoV-2 lineages among infections.

†††† *Region 1*: Connecticut, Maine, Massachusetts, New Hampshire, Rhode Island, and Vermont; *Region 2*: New Jersey, New York, Puerto Rico, and U.S. Virgin Islands; *Region 3*: Delaware, District of Columbia, Maryland, Pennsylvania, Virginia, West Virginia; *Region 4*: Alabama, Florida, Georgia, Kentucky, Mississippi, North Carolina, South Carolina, and Tennessee; *Region 5*: Illinois, Indiana, Michigan, Minnesota, Ohio, and Wisconsin; *Region 6*: Arkansas, Louisiana, New Mexico, Oklahoma, and Texas; *Region 7*: Iowa, Kansas, Missouri, and Nebraska; *Region 8*: Colorado, Montana, North Dakota, South Dakota, Utah, and Wyoming; *Region 9*: American Samoa, Arizona, California, Guam, Hawaii, Marshall Islands, Nevada, Northern Mariana Islands, Federated States of Micronesia, and Palau, *Region 10*: Alaska, Idaho, Oregon, and Washington.

§§§§
https://covid.cdc.gov/covid-data-tracker/#trends_dailycases

¶¶¶¶
https://www.biorxiv.org/content/10.1101/2021.12.31.474032v1

*****
https://www.medrxiv.org/content/10.1101/2021.12.30.21268495v1.full.pdf

††††† https://papers.ssrn.com/sol3/papers.cfm?abstract_id=3996320

§§§§§ S-gene target failure: widely used TaqPath COVID-19 Combo Kit polymerase chain reaction assay used for screening and as a proxy for Alpha and Omicron variant detection.
