# *Erratum*: Vol. 71, No. 6

**DOI:** 10.15585/mmwr.mm7114a4

**Published:** 2022-04-08

The report “Genomic Surveillance for SARS-CoV-2 Variants: Predominance of the Delta (B.1.617.2) and Omicron (B.1.1.529) Variants — United States, June 2021–January 2022” contained several errors.

On page 206, the list of authors should have read, “Anastasia S. Lambrou, PhD^1,2,^*; Philip Shirk, PhD^1,^*; Molly K. Steele, PhD^1^; Prabasaj Paul, PhD^1^; Clinton R. Paden, PhD^1^; Betsy Cadwell, MSPH^1^; Heather E. Reese, PhD^1^; Yutaka Aoki, PhD^1^; Norman Hassell, MS^1^; **Jason Caravas, PhD^1,3^; Nicholas A. Kovacs, PhD^1,4^; Jonathan G. Gerhart, MS^5^; Han Jia** **Ng, PhD^1,6^; **Xiao-yu Zheng, PhD**^1,7^**; **Andrew Beck, PhD^1^; Reina Chau, MS^1^; Roxana Cintron, MS^1^; Peter W. Cook, PhD^1^; Christopher A. Gulvik, PhD^1^; Dakota Howard^1^; Yunho Jang, PhD^1^; Kristen Knipe, MS^1^; Kristine A. Lacek, MS^1^; Kara A. Moser, PhD^8^; Adrian C. Paskey, PhD^1^; Benjamin L. Rambo-Martin, PhD^1^; Roopa R. Nagilla, MS^7^; Adam C. Retchless, PhD^1^; Matthew W. Schmerer, PhD^1^; Sandra Seby, MS^1^; Samuel S. Shepard, PhD^1^; Richard A. Stanton, PhD^1^; Thomas J. Stark, PhD^1^; Anna Uehara, PhD^1^; Yvette Unoarumhi, MS^1,4^; Meghan L. Bentz^1^; Alex Burgin^1^; Mark Burroughs^1^; Morgan L. Davis, MS^1,5^; Matthew W. Keller, PhD^1^; Lisa M. Keong^7^; Shoshona S. Le^1^; Justin S. Lee, DVM, PhD^1^; Joseph C. Madden Jr, PhD^1^; Sarah Nobles, MS^1^; D. Collins Owuor, PhD^1^; Jasmine Padilla^1,5^; Mili Sheth, PhD^1^; Malania M. Wilson, MS, MBA^1^;** Sarah Talarico, PhD^1^; Jessica C. Chen, PhD^1^; M. Steven Oberste, PhD^1^; Dhwani Batra, MS, MBA^1^; Laura K. McMullan, PhD^1^; Alison Laufer Halpin, PhD^1^; Summer E. Galloway, PhD^1^; Duncan R. MacCannell, PhD**^3^**; Rebecca Kondor, PhD^1^; John Barnes, PhD^1^; Adam MacNeil, PhD^1^; Benjamin J. Silk, PhD^1^; Vivien G. Dugan, PhD^1^; Heather M. Scobie, PhD^1^; David E. Wentworth, PhD^1^.”

On pages 210–11 the acknowledgments should have read, “**Eric Chirtel, Victoria Caban Figueroa, Tymeckia Kendall, Garrett Longmire, Brian Mann, Nicole Patterson, Catherine Smith, Erica Sula, Subblakshmi Voleti, Jonathan Zhong.**”

On page 211, the author affiliations should have read, “^1^CDC COVID-19 Emergency Response Team; ^2^Epidemic Intelligence Service, CDC; **^3^**Office of Advanced Molecular Detection, National Center for Emerging and Zoonotic Infectious Diseases, CDC; **^4^Oak Ridge Institute for Science and Education, Oak Ridge, Tennessee; ^5^ASRT** **Inc., Smyrna, Georgia; ^6^Eagle Global Scientific, LLC, San Antonio, Texas;** **^7^**General Dynamics Information Technology, Inc., Falls Church, Virginia; **^8^Goldbelt C6, LLC, Chesapeake, Virginia.**”

All authors have completed and submitted the International Committee of Medical Journal Editors form for disclosure of potential conflicts of interest. No potential conflicts of interest were disclosed.

